# Supplementary material for: Extracellular Traps Released by Neutrophils from Cats are Detrimental to Toxoplasma gondii Infectivity
Source: Microorganisms. 2020 Oct 22;8(11):1628. doi: 10.3390/microorganisms8111628 (PMC7716220; doi:10.3390/microorganisms8111628)
Supplement: Supplementary file 1 [file microorganisms-08-01628-s001.zip › Supplementary Files_20201012.docx]

Supplementary material for “Extracellular Traps Released by Neutrophils from Cats are Detrimental to *Toxoplasma gondii* Infectivity”

Isabela S. Macedo ^1,†^, Marcos V. A. Lima ^1,†^, Jéssica S. Souza ^1^, Natalia C. Rochael ^2^, Pedro N. Caldas ^3^, Helene S. Barbosa ^1^, Flávio A. Lara ^4^, Elvira M. Saraiva ^2^ and Rafael M. Mariante ^1,^*

^1^ Laboratório de Biologia Estrutural, Instituto Oswaldo Cruz, Fiocruz, Rio de Janeiro 21040-360, RJ, Brazil; macedos.isa@gmail.com (I.S.M.); marquinhos9796@gmail.com (M.V.A.L.); jsouza.biomed@gmail.com (J.S.S.); helene@ioc.fiocruz.br (H.S.B.)

^2^ Laboratório de Imunobiologia das Leishmanioses, Instituto de Microbiologia Paulo de Góes, Universidade Federal do Rio de Janeiro, Rio de Janeiro 21941-902, RJ, Brazil; natyrochael@yahoo.com.br (N.C.R.); esaraiva@micro.ufrj.br (E.M.S.)

^3^ HVN Hospital Veterinário Niterói, Niterói 24360-440, RJ, Brazil; pedrovet@pedrovet.com.br

^4^ Laboratório de Microbiologia Celular, Instituto Oswaldo Cruz, Fiocruz, Rio de Janeiro 21040-360, RJ, Brazil; flavioalveslara2000@gmail.com

^†^ These authors contributed equally to this work.

***** Correspondence: [rafael.mariante@ioc.fiocruz.br](mailto:rafael.mariante@ioc.fiocruz.br) or rafaelmariante@gmail.com; Tel.: +55-21-2562-1018


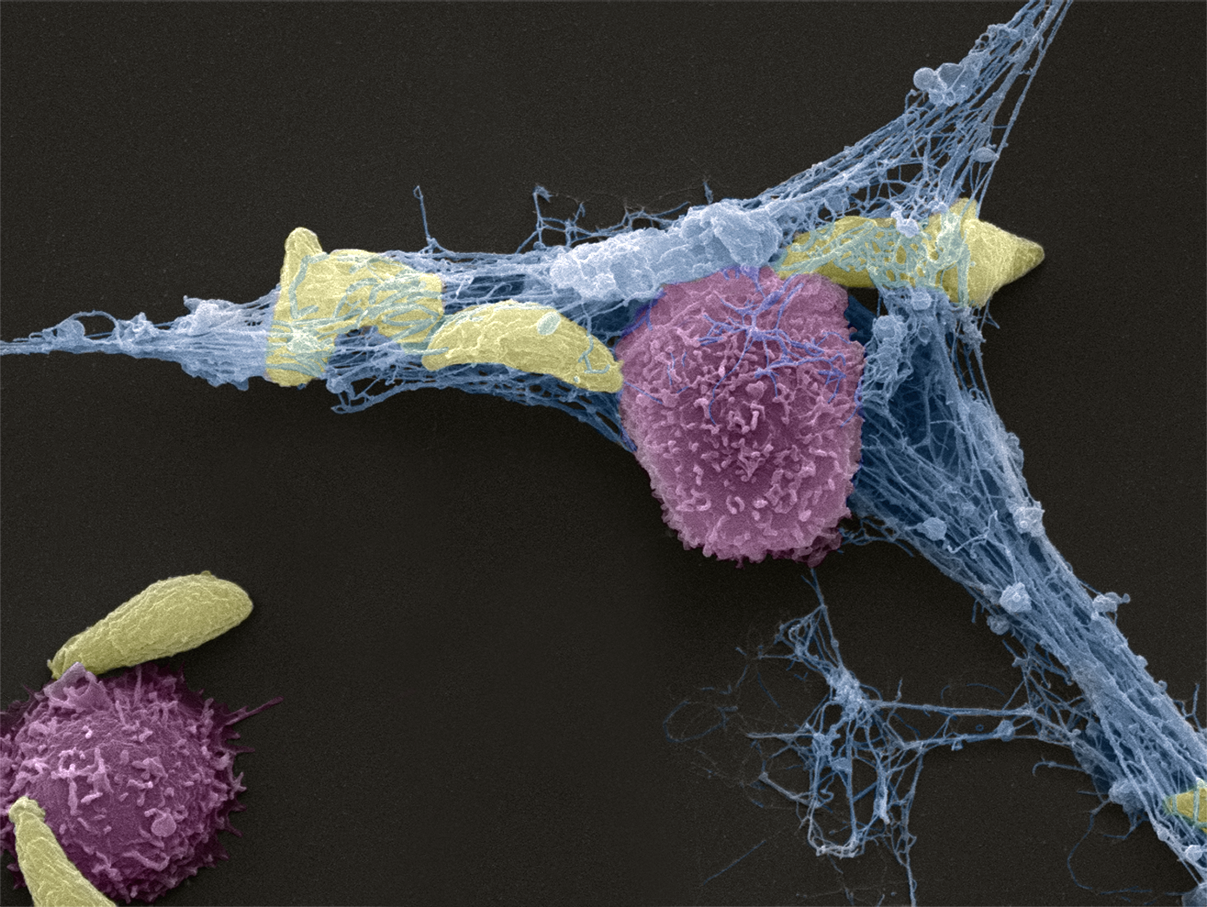


**Supplementary Figure S1**

**Supplementary Figure S1.** Neutrophils from cats produce NET in response to PMA and *Leishmania amazonensis*. Neutrophils from different donors were incubated for 180 min with either (**A**) PMA (100 nM) or (**B**) promastigotes of *L. amazonensis* (LA; 5:1 parasites:neutrophil ratio). Supernatants were collected and released dsDNA was quantified with PicoGreen Kit (*n* = 7-11). Results are shown as means ± SD. * *p* < 0.05; ** *p* < 0.01.

**Supplementary Figure S2**

**Supplementary Figure S2.** Release of classical NET-derived DNA in response to *T. gondii* is independent of MPO, PAD, PI3Kγ and MEK. Neutrophils were pre-treated or not with (**A**) the myeloperoxidase inhibitor I (MPOi; 600 nM), (**B**) the PAD inhibitor chloroamidine (Cl-A; 12 μM), (**C**) the PI3Kγ selective inhibitor AS605240 (AS60; 10 µM) or (**D**) the MEK inhibitor PD98059 (PD98; 60 µM) for 30 min and then stimulated for 180 min with RH tachyzoites (5:1 parasites:neutrophil ratio). Supernatants were collected and released dsDNA was quantified with PicoGreen Kit (n = 10-12). Results are shown as mean (SD). ** *p* < 0.01; *** *p* < 0.001.

**Supplementary Figure S3**

**Supplementary Figure S3.** Neutrophil viability after treatment with inhibitors. The cytotoxicity of the inhibitors to neutrophils was examined with PrestoBlue Cell Viability Reagent. After treatment with the compounds, PrestoBlue was added 20 min before the end of the incubation time. Analysis was performed in a microplate reader using 560/590 nm excitation/emission wavelengths (*n* = 5). The data are represented as percentage of control and are shown as mean (SD).

**Supplementary Figure S4**

**Supplementary Figure S4.** Spontaneous death of *Toxoplasma gondii* in culture medium. The parasites were seeded in glass culture dishes and kept at 37°C for up to 5 hours in the presence of propidium iodide (PI). At the indicated times, some samples were collected and cell death was evaluated (*n* = 2). The data are represented as a percentage of cells that incorporated PI and are shown as mean (SD).

**Supplementary Figure S5**


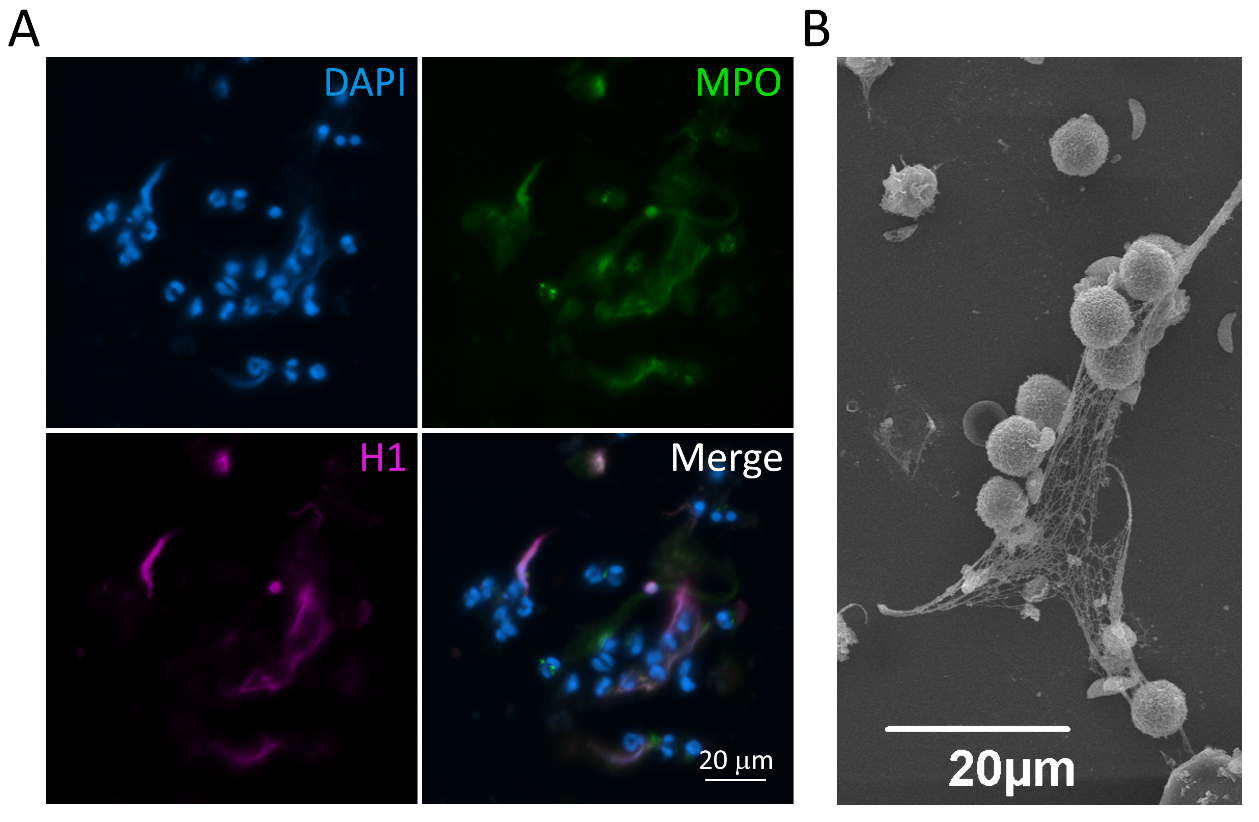


**Supplementary Figure S5.** NET formation induced by *Toxoplasma gondii* tachyzoites. Neutrophils were incubated for 180 min with RH strain tachyzoites (5:1 parasites:neutrophil ratio), fixed and stained for myeloperoxidase (MPO) and histone H1 (**A**) or fixed and processed for scanning electron microscopy (**B**). Note the small clusters of NET aggregating neutrophils.

**Supplementary Videos**

**Supplementary Video S1.** Time lapse video showing the release of NET by cat neutrophils in response to *T. gondii*. Neutrophils were stimulated with *T. gondii* tachyzoites (5:1 parasite:neutrophil ratio) in the presence of propidium iodide (PI). Sequential images begun to be recorded after 15 min of interaction. Arrows point to neutrophils releasing NET after about 80 min of interaction with parasites.

**Supplementary Video S2.** Tachyzoites of *T. gondii* dying in contact with cat NET. Neutrophils were stimulated with *T. gondii* tachyzoites (5:1 parasite:neutrophil ratio) in the presence of propidium iodide (PI). Images in the sequence begun to be recorded after 135 min of interaction. Arrows point to two parasites dying after contact with NET.
