# Supplementary figures and images for: Extracellular Traps Released by Neutrophils from Cats are Detrimental to Toxoplasma gondii Infectivity
Source: Microorganisms. 2020 Oct 22;8(11):1628. doi: 10.3390/microorganisms8111628 (PMC7716220; doi:10.3390/microorganisms8111628)

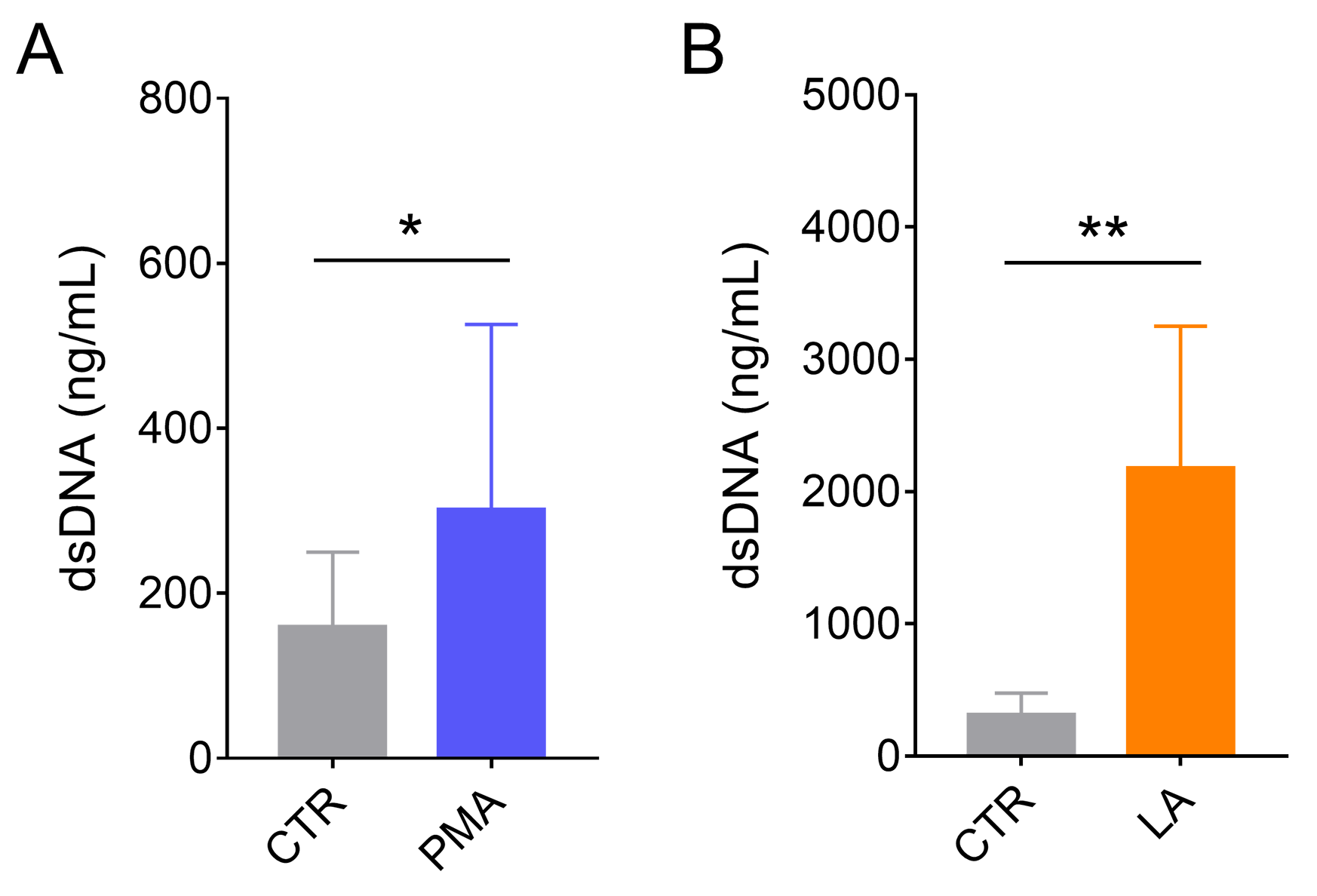

Supplement: Supplementary file 1 [file microorganisms-08-01628-s001.zip › Supplementary Figure_1.tif]

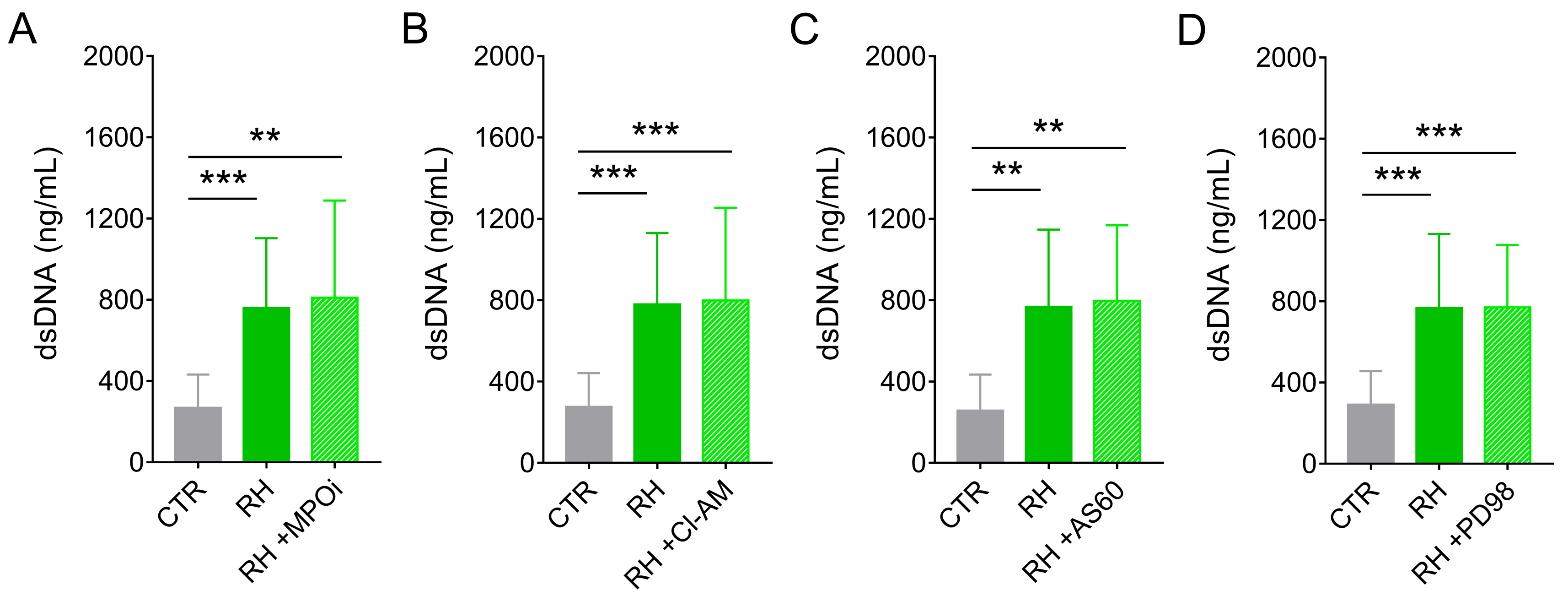

Supplement: Supplementary file 1 [file microorganisms-08-01628-s001.zip › Supplementary Figure_2.tif]

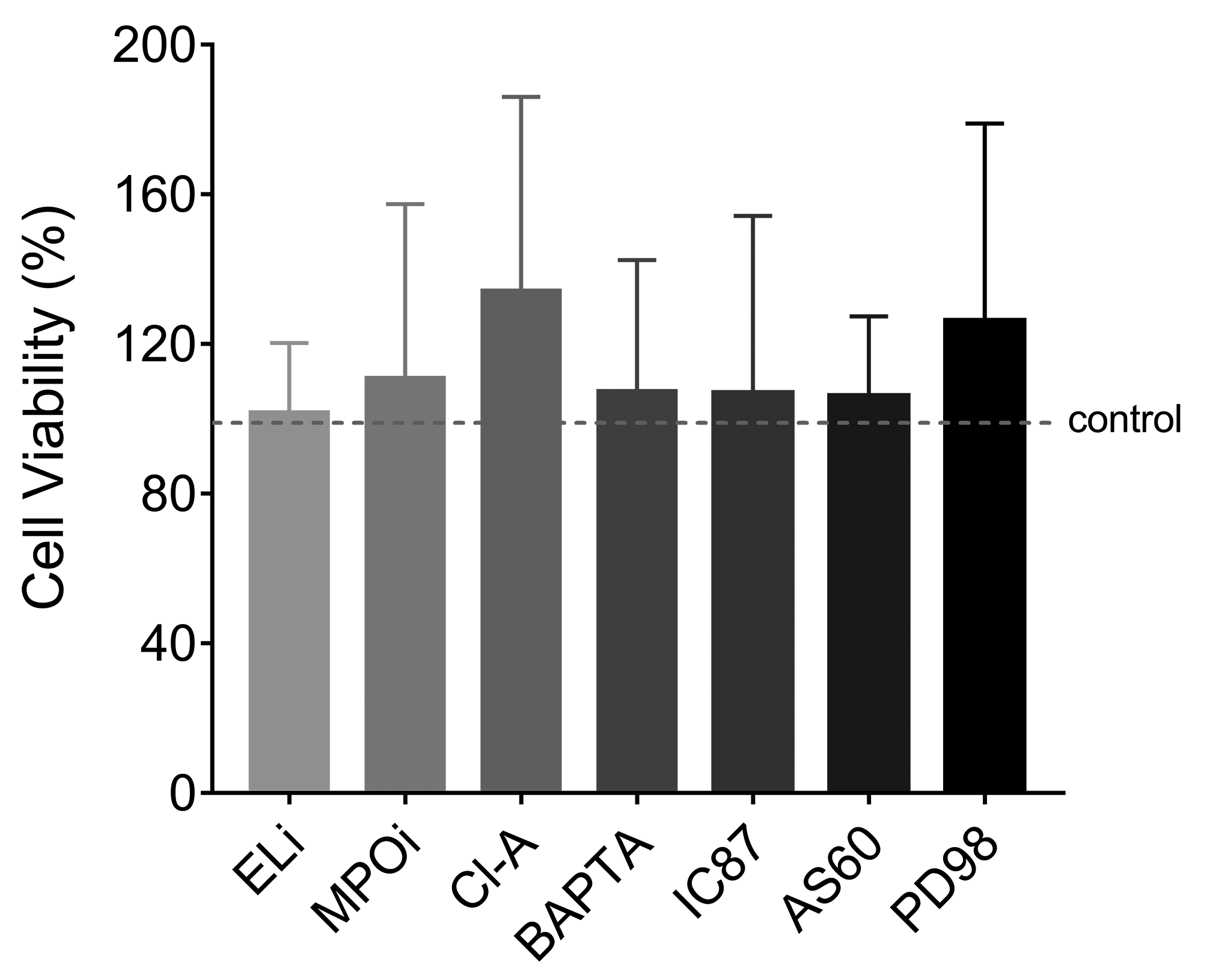

Supplement: Supplementary file 1 [file microorganisms-08-01628-s001.zip › Supplementary Figure_3.tif]

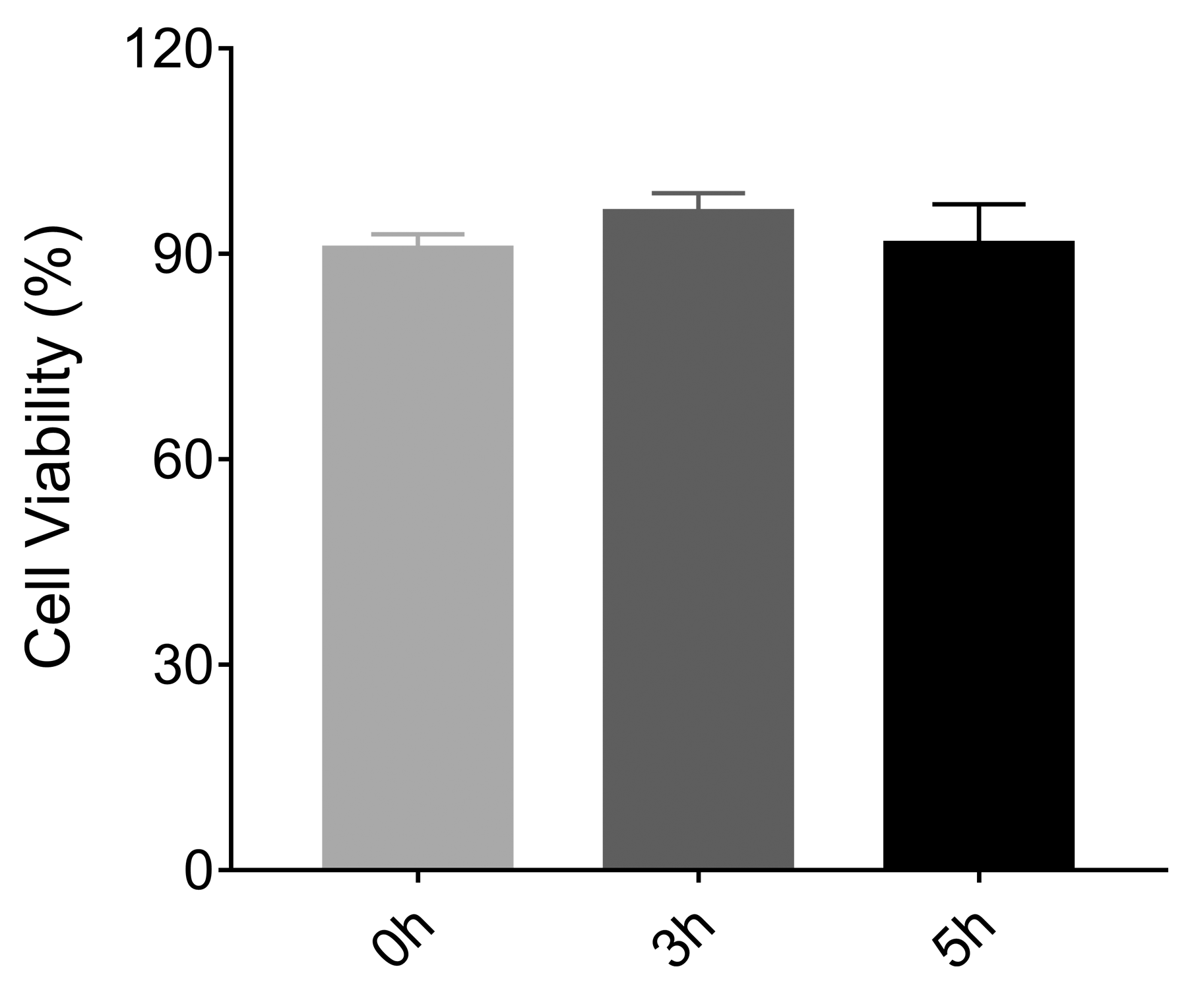

Supplement: Supplementary file 1 [file microorganisms-08-01628-s001.zip › Supplementary Figure_4.tif]

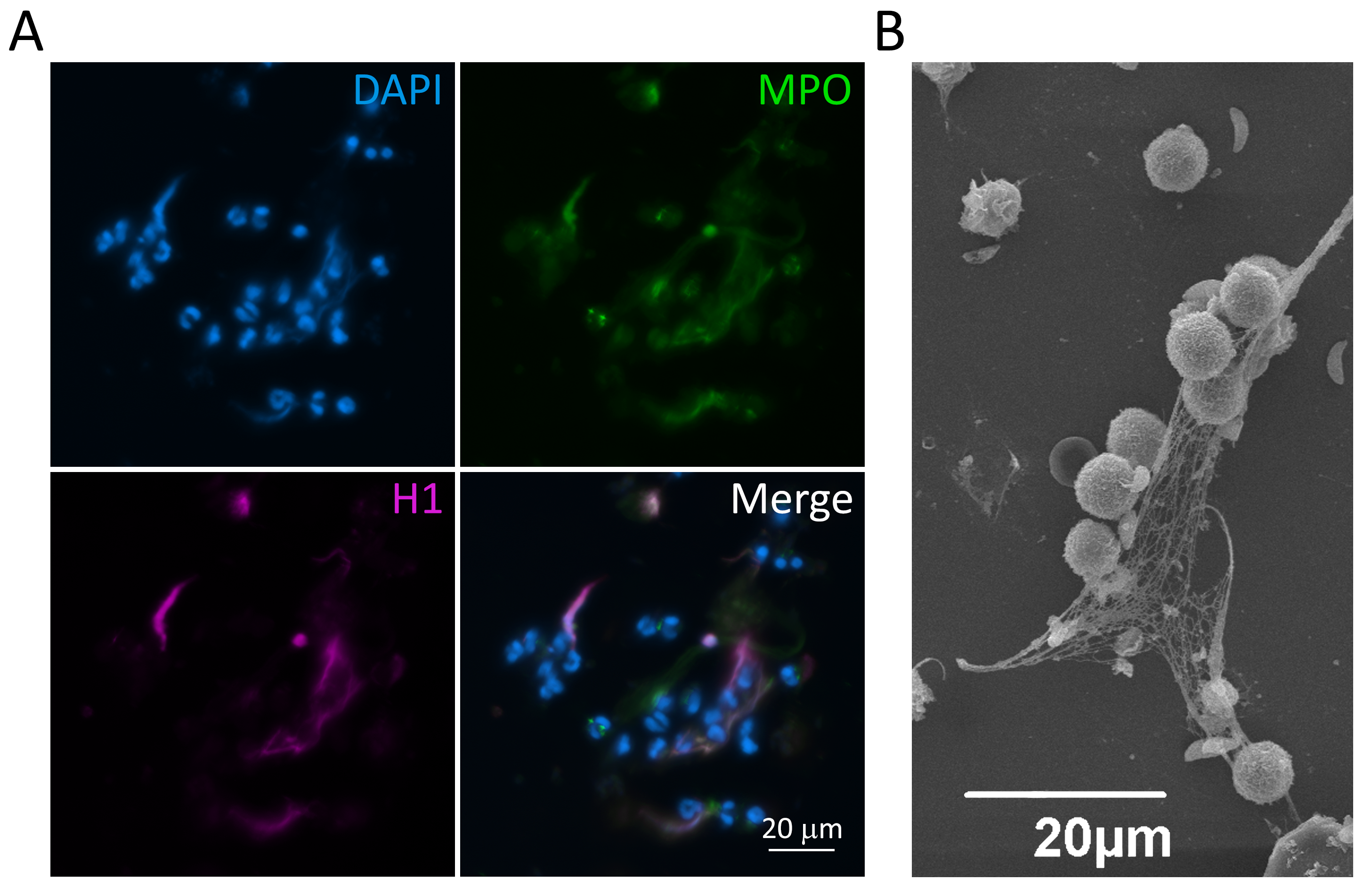

Supplement: Supplementary file 1 [file microorganisms-08-01628-s001.zip › Supplementary Figure_5.tif]

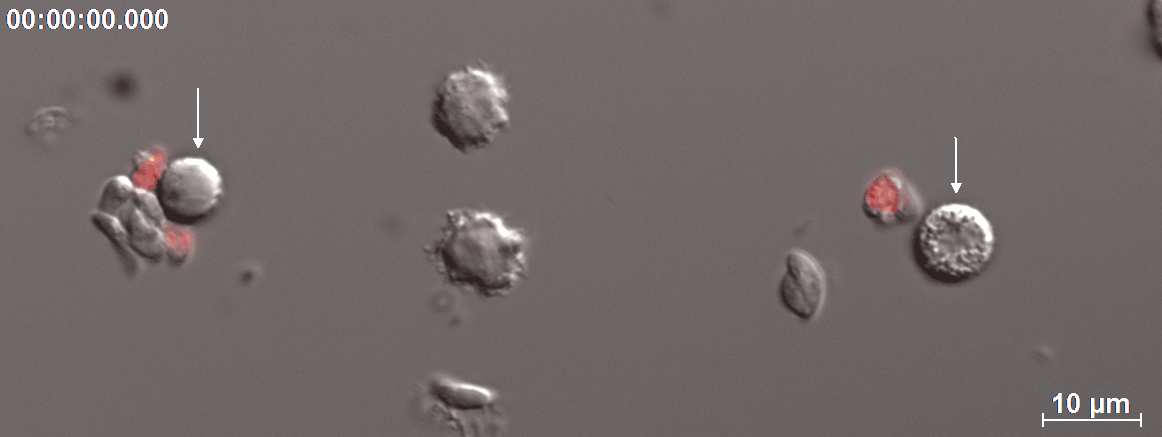

Supplement: Supplementary file 1 [file microorganisms-08-01628-s001.zip › Supplementary Video_1.gif]

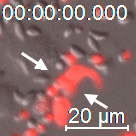

Supplement: Supplementary file 1 [file microorganisms-08-01628-s001.zip › Supplementary Video_2.gif]
